# Organ Markets, Options, and an Over-Inclusiveness Objection: On Rippon’s Argument

**DOI:** 10.1007/s11673-024-10363-x

**Published:** 2024-08-29

**Authors:** J. Damgaard Thaysen, J. Sønderholm

**Affiliations:** Department of Culture and Learning, Aalborg University, Aalborg Ø, Denmark

**Keywords:** Bioethics, Organ sale, Exploitation, Harm to the poor, Limits of markets

## Abstract

Human organs available for transplant are in short supply. One way to increase the supply of organs consists in legalizing a live donor market. Such a market is, however, controversial. This article is about an objection to live donor organ markets made by Simon Rippon. Rippon’s objection is that the presence of a market option creates new social and legal pressures that harm the poor. Legalizing the option of selling your organs transforms into a harmful, and morally indefensible, social, and legal pressure to sell on the financially desperate. This article defends the conclusion that Rippon’s argument fails as an objection to live donor organ markets. It fails because it has implausibly expansive implications about which markets are morally problematic. In short, Rippon’s argument proves too much. Sections one and two introduce Rippon’s argument. Sections three and four contain the argument against Rippon. The main argumentative move is that the features of an organ market that, according to Rippon, justify a ban on such a market are features that also characterize several other markets that are normally considered unproblematic, for example, markets where individuals sell their labour abroad in jobs that are dangerous. So, if an organ market should be legally impermissible, so should these labour markets. Section five considers several objections to the argument against Rippon. It is argued that these objections fail. Section six is a conclusion that sums up the findings of the article.

## Introduction

Kidneys, and other organs, available for transplant are in desperately short supply. In the United States, 103,223 individuals are on the national transplant waiting list and seventeen people die each day waiting for an organ transplant (see Human Resources and Services Administration 2024). One way to increase the supply of kidneys (and other organs) consists in legalizing a live donor market. One virtue of a well-functioning market in a particular is that the market allocates resources effectively. In such a market, rational individuals will trade with one another only when both parties benefit from the trade, and there will be a continual adjustment of supply and demand that, eventually, leads to a situation in which there is no excess supply and no excess demand. So, given the high demand for kidneys, it is likely that this demand, in a well-functioning market, will be met with an increased supply of kidneys (see Satz 2010, 17–18, 191-92; Brennan and Jaworski 2016, 8).

Yet, a legal live donor market is a controversial suggestion (see Greasley 2014; Albertsen 2020; Rippon 2017; Satz 2010, 191–202). This article is about an objection to live donor organ markets made by Simon Rippon (Rippon 2014). The objection goes as follows: the presence of a market option creates new social and legal pressures and subjecting the poor to such pressures harms them. Individuals are sometimes socially and legally expected to avail themselves of whatever legal opportunities to raise funds they have. In particular, financially struggling individuals may be required to exhaust their opportunities for providing for themselves, by selling any valuable assets they have and taking any available employment, before becoming eligible for public assistance or bankruptcy protection. If selling organs were legal, then, e.g., kidneys would become a valuable economic asset that individuals could be required to sell before becoming eligible for bankruptcy protection or public assistance. You cannot normally declare yourself bankrupt *and* keep your Ferrari and owning it will count against any means-tested public benefit. Legalize live donor organ markets and possessing two healthy kidneys becomes much like owning a Ferrari. Thus, legalizing the option of selling your kidney would transform into a harmful, and morally indefensible, social and legal pressure to sell on the financially desperate (Rippon 2014, 147–148).

This article concludes that Rippon’s argument fails as an objection to live donor organ markets. It does so because it has implausibly expansive implications about which markets are morally problematic. In short, Rippon’s argument proves too much. Our critical discussion of Rippon’s argument is not a general defence of organ markets, since other objections to organ markets may be raised. Nevertheless, a critical discussion of the plausibility of Rippon’s argument is of significant interest to the general discussion about organ markets for reasons we outline immediately below.^1^

## The Special Badness of Market-Driven Social and Legal Pressure to Sell Organs

There is much to admire about Rippon’s innovative argument. The argument deftly avoids paternalism; it does not claim that organ markets are problematic, because potential organ sellers will choose poorly but that having the option of selling one’s kidney harms the poor, irrespective of whether they choose to sell. Nor is the argument based on a moralistic claim about the intrinsic wrongfulness of selling one’s organs (Rippon 2014, 146–147). This makes the argument easier to reconcile with the traditional liberal aversion to paternalism and moralism (Cf. Feinberg 1987, 14–16).

As Rippon is aware, his argument seems *prima facie* to apply to every market. The possibility of selling anything can lead to some people facing social and legal pressure to sell it. As Rippon does not object to the market economy as such, he attempts to explain why his argument renders live donor organ markets unjust but does not show that other, less controversial, markets are unjust. Rippon concedes that his argument shows that there is something bad about the social and legal pressure to sell accompanying any market but this badness is outweighed by the general benefits of a market economy (Rippon 2014, 149). Rippon argues that, though selling organs is not intrinsically wrong, “two features together […] explain the special badness of market-driven social and legal pressure to sell organs” (Rippon 2014, 149). These two features are:

A) The market involves “small, but not insignificant life-changing risks” (Rippon 2014, 149) that are either i) not justified to impose by other considerations *or* ii) not fairly distributed by market forces.
B) Market-driven social and legal pressure to sell organs violates a right to have “fully autonomous veto control over any physical incursions on the intimate parts of our bodies by other people” (Rippon 2014, 149).

To succeed A) and B) must distinguish live donor organ markets from all permissible markets. Below we argue that A) and B) apply to several mainstream and *prima facie* permissible markets. If we are right about this, Rippon’s argument is overinclusive. Of course, the fact that A) and B) apply to some *prima facie* permissible market could be taken as evidence that this market is not permissible after all. This move is plausible enough the first couple of times, especially if the markets in question are controversial and/or obscure. However, as the markets to which features A) and B) arguably apply become more and more mainstream, it becomes less plausible to defend Rippon's argument by simply conceding that these arguments too are unjust."

Note also that it is unclear how “*together”* modifies Rippon’s claim about the connection between A) and B) and the special badness of market-driven social and legal pressure. We take it to mean that A) and B) are individually insufficient but jointly sufficient conditions of *special badness.*

## On the Similarity of Selling Your Kidney and Selling Your Labor in a Dangerous Job

Consider the following:

Miguel is a citizen of Acapulco, Mexico. Miguel is unemployed and in financially dire straits. There is demand for agricultural workers to tend crops in California. Getting to California involves a long journey, and Miguel will have to stay in California for an extended period, if he takes on work as an agricultural worker in crop production. Current U.S. immigration legislation makes it illegal for Miguel to work in the U.S. without a permit.

We contend that Rippon’s argument implies that the members of congress who support the current U.S. Immigration Policy and the Immigration and Customs Enforcement (ICE) agents that enforce it are doing Miguel and poor Latin Americans like him a favour by shielding them from especially bad “market-driven social and legal pressure to sell” (Rippon 2014, 149) their labour in a dangerous job, in a faraway place by keeping this option off the table. This is a striking implication to say the least. If the option of taking a job as an agricultural worker in the United States were legalized, the presence of this new option would cause Miguel to be subjected to “the social and legal costs to pay for failing to take employment, when you are able to do so” (Rippon 2014, 148). For instance, he may no longer be eligible for unemployment benefits and his social network might be less inclined to help him, now that he can provide for himself by taking a job as an agricultural worker in California. The remainder of the paper is dedicated to demonstrating this. To do so, we need to show that taking a job as an agricultural worker in a far-off place, satisfies A) and B).

Let us start by examining A). “Small but not insignificant life-changing risk” is not an exact term. But we can use the risks involved in a live organ donation, a 1 in 3000 mortality rate, as a benchmark (Rippon 2014, 149). Similarly, it is hardly self-evident what exactly constitutes an all-things-considered sufficient justification of imposing a risk, though Rippon helpfully, exemplifies with a situation “if the country is fighting a just war and conscripts to the armed forced are necessary” (Rippon 2014, 149). Rippon’s remark that it “does not seem fair that allocating these risks by way of market forces that de facto place nearly all the pressure to take them on those in poverty” (Rippon 2014, 149), tells us enough about what counts as a fair distribution of risks for present purposes.

Working as an agricultural worker involves small but not insignificant life-changing risks. United States agricultural workers in crop production had a roughly 1 in 4800 annual risk of dying on the job in 2020.^2^ While the yearly chance of dying on the job for a U.S. worker in crop production is not quite as high as the one involved in a nephrectomy (the surgical removal of a kidney), a nephrectomy occurs only once, while work in crop production is not limited to one year. The risk of *two years* of work in crop production exceeds the risk of a nephrectomy. So, it seems fair to see the mortality rates as at least comparable. Work in crop production is only one among several professions with a risk profile comparable to that of a nephrectomy. These professions include being a logger, roofer, garbage collector, delivery driver, iron and steel worker, underground mining machine operator, construction equipment operator, construction helper, construction labourer, grounds maintenance worker, landscaper, and power line installer. The mortality rate of *two* years’ work in *any* of these professions exceeds the mortality rate of a nephrectomy (U.S. Bureau of Labor Statistics 2020).

There is little reason to think that market forces will allocate the significant risk of working in any of these jobs any more fairly than the significant risk of undergoing nephrectomy. These professions are low prestige, and individuals are unlikely to seek these jobs for other reasons than financial need. Thus, the market for selling one’s labour as, say, an agricultural worker in crop production, construction helper, grounds maintenance worker, etc. satisfies A) no less than selling a kidney.

## On Interference With Our Veto Control Over What Happens to Our Body

We now turn to B). Our main line of argument shall be to raise several problems regarding B) and show that when these problems are solved several *prima facie* unproblematic markets satisfy B). In a key passage of his formulation of B) Rippon invokes:

… the peculiar importance to human beings of our having fully autonomous veto control over any physical incursions on the intimate parts of our bodies by other people. It is psychologically important to us that our negative right to exclude others from interfering with our physical bodies is protected. (Rippon 2014, 149).

Rippon makes two claims that are not identical. The assertion that:

1. It is important to have fully autonomous veto control over any physical incursion on the intimate part of our bodies by other people.

is *far more specific* than the assertion that:

2) It is important to respect our negative right to exclude others from interfering with our physical bodies.

There are several ways to interfere with someone’s physical body, that do not involve incursions on the *intimate* parts, e.g., cutting someone’s nails without consent. The highly specific formulation of 1) suggests that the normative force of 1) is explained by a more general principle, such as 2). It would be surprising if a principle as specific as 1) were normatively basic. The relationship between 1) and 2) seems best understood as akin to the relationship between, say, “it is wrong to hit people with your fist on a rainy Tuesday for no reason” and “harming others is pro tanto wrong.” The former is true but is clearly explained by some more abstract principle like the latter. Consider, moreover, the normative judgments Rippon presents as warrant for B), namely: the principle of consent for medical interventions, the special badness of rape, the special badness of some forms of physical torture, and what is wrong with state-mandated redistribution of organs from live donors (Rippon 2014, 149). All these normative judgements are as easily explained by 2) as by 1). In fact, it is unclear that there is any rational basis for valuing 1) without also valuing 2). We are not contending that B) lacks a rational basis but that B) is best motivated by a general concern with exclusive control over what happens to our body. Given that Rippon himself slides between 1) and 2), it is unclear whether he would disagree. Could he not simply concede this point?

No. If Rippon concedes that we are concerned with B) because we are concerned with general control over what happens to our body, his view implies that creating the option of taking a dangerous job, far away impermissibly subjects the financially struggling to a market-driven legal and social pressure to sell. This is what the story of Miguel illustrates. Working in crop production is quite risky**,** so taking the job certainly has a small, but not insignificant life-changing risk. If Miguel takes on work in crop production in California, he will have to relocate his body more than 4000 km north. Thus, the market-driven social and legal pressure that would result from giving him this option, infringes on his negative right to veto interference with his physical body in a manner analogous to the consequences of giving Miguel the option of selling his kidney. Therefore, both criteria for the special badness market-driven pressure are met. If Rippon thinks that legalizing a live donor market is impermissible, he is committed to believing that opening labour markets in low prestige, dangerous jobs to far away foreigners is equally impermissible.

The current U.S. immigration regime is controversial, and its proponents do not normally defend it on the grounds that it benefits the would-be immigrants. Yet this is what Rippon’s argument implies. More generally, Rippon’s argument implies that the option of finding employment abroad (or across a sufficiently large and diverse country) subjects the poor to market-driven social and legal pressure to relocate in a way that violates their right to control what happens to their body. When the job in question also involves significant life-changing risks, as the job as agricultural worker does, the argument implies that legally permitting a market in foreign labour is unjust.

## Objections

Several objections can be made to the story of Miguel, but as far as these arguments have bite against the Miguel example, they are equally effective at undermining Rippon’s argument. First, one might object that Miguel is not actually coerced to do anything but merely presented with an option possibly accompanied by some expectation that he will take it. However, this is also true of the legalization of live donor organ markets. The type of harm Rippon associates with these markets do not arise from the sellers being coerced in any strict sense but “merely” from sellers experiencing pressure. At any rate, the type of pressure involved—social and legal expectations—are identical in both examples. Note also, that what Miguel is being pressured to do, move his physical body to a specific place for an extended period, is the kind of thing that is important to retain control over. If Miguel were strictly coerced to relocate in this manner, it would be kidnapping, which is a serious violation of Miguel’s right to exclusive control over interference with his body. So, anyone who denies that Miguel’s right to control his body is violated by the market-driven social and legal pressure to relocate would have to do so because “mere” social and legal pressure is not enough to violate a right to control our own body. That may be, but that would undermine Rippon’s argument.

Second, one might argue that legalizing the option of working in crop production in the United States will not make a difference to the legal and social pressure experienced by Miguel. One might think this for two reasons, namely; C) the option of travelling to California to work is available and salient regardless of the legality of taking this option. There might be social pressure to take the illegal, but well-known, option to work illegally in California; D) there might be little social and legal pressure to take the option of travelling to California to work even if it is legal. For instance, state agencies can require people to take employment when they are able to do so to be eligible for unemployment benefits but exempt some legal income-generating option line of work from consideration when assessing whether someone is willing to work. We have a great deal of sympathy for these objections, but again they have equal force against Rippon’s argument. Indeed, Dworkin pushes an objection identical to D), when he points out that it is not clear whether there is any significant social and legal pressure on women to sell their eggs to infertile couples, even though this option is legal in the United States (Dworkin 2014, 151).

Third, an exasperated reader might be tempted to exclaim that organs and the physical intrusion upon our body are special. It is true that the story of Miguel involves no penetration of the body. However, it remains unclear whether the badness of physical intrusions on the body is distinct from the badness of violations of our right to control what happens to our body more generally. After all, the view that the badness of physical incursions is distinct, would seem to commit us to the view that it is *pro tanto* worse to be stabbed to death than tied up and starved to death. Rippon himself slides between “fully autonomous veto control over any physical incursions on the intimate parts of our bodies by other people,” the “negative right to exclude others from interfering with our bodies,” and “veto control over physical intrusions upon our bodies by others” (Rippon 2014, 149). Any of these three assertions can explain the normative judgments, about our insistence on the principle of consent for medical interventions, the badness of rape and the dreadfulness of certain forms of physical torture, he uses as warrant. The strong point in favour of seeing the badness of physical incursions on the intimate parts of our bodies as distinct is probably the special badness of rape. However, the force of this judgment can only be connected to the sale of organs through equivocation about the meaning of “intimate.” Either “intimate parts” refers narrowly to male and female sexual organs. Alternatively, the term is to be understood more broadly and refers to internal organs such as kidneys, the heart, lungs, and the liver. On the first meaning, the passage about “…veto control over any physical incursions on the intimate parts of our bodies…” is a neat explanation of the special badness of rape but cannot be used in an argument against a live donor organ market, since kidneys etc. are not intimate parts. On the second meaning, the passage is of relevance to live donor organ markets but cannot be used to explain why we find rape “a particularly appalling crime.” In typical examples of rape, there is no physical incursion on the victim’s kidneys, heart, lungs, or liver.

## Conclusion

Rippon’s argument against organ markets implies that it should be legally impermissible to sell your labour abroad in a job with not insignificant professional risk, such as agricultural worker in crop production, logger, roofer, garbage collector, delivery driver, iron and steel worker, underground mining machine operator, construction equipment operator, construction helper, construction labourer, grounds maintenance worker, landscaper, and power line-installer. These are some striking revisionary implications, not least for immigration policy. Perhaps Rippon, and those positively inclined towards his argument, find these implications unpalatable. If so, they should reconsider whether Rippon’s innovative argument justifies banning live donor organ markets. That said, our reply to Rippon does not amount to a positive case in favour of organ markets, since there are many other potential objections to organ markets.

In ending, we should stress that we believe Rippon is on to something. We see no reason to doubt the claim that making a new market option available to the poor will create social and legal pressure to take that option. That this is at least somewhat bad in many cases is also plausible. However, Rippon’s argument fails as an argument against live donor organ markets in particular. We suggest that what the argument really points to is a problematic feature of the practice of means-testing as a method for determining whether someone qualifies for financial assistance. Namely, that means-testing creates a legal and social pressure to sell, whatever one is allowed to sell on the market.

## Data Availability

Not applicable.

## Footnotes

1 It should be noted that we are not the first to critically discuss Rippon’s article. In particular, commentaries by Walsh, Radcliffe-Richards, and Dworkin (Walsh 2014; Dworkin 2014; Radcliffe-Richards 2014) were published along with the original article. Many interesting points were made in these contributions, but none of the commentaries raised the worry about the over-inclusiveness of Rippon’s argument that this paper is focused on.

2 United States workers in crop production were fatally injured at a rate of 20.8 pr. 100,000 full-time equivalent workers in 2020 (U.S. Bureau of Labor Statistics 2020).

## References

[CR1] Albertsen, A. 2020. If the price is right: The ethics and efficiency of market solutions to the organ shortage. *Journal of Bioethical Inquiry* 17(3): 357–367. 32557218 10.1007/s11673-020-09981-y

[CR2] Brennan, J., and P. Jaworski (eds). 2016. *Markets without limits: Moral virtues and commercial interests*. New York, London: Routledge, Taylor & Francis Group.

[CR3] Dworkin, G. 2014. Organ sales and paternalism. *Journal of Medical Ethics* 40(3): 151–152. 22718855 10.1136/medethics-2012-100647

[CR4] Feinberg, J. 1987. *The moral limits of the criminal law volume 1: Harm to others*. Oxford University Press.

[CR5] Greasley, K. 2014. A legal market in organs: The problem of exploitation. *Journal of Medical Ethics* 40(1): 51–56. 23001920 10.1136/medethics-2012-100770

[CR6] Human Resources and Services Administration. 2024. Organ donation statistics. https://www.organdonor.gov/learn/organ-donation-statistics. Accessed March 18, 2024.

[CR7] Radcliffe-Richards, J. 2014. Commentary by Janet Radcliffe-Richards on Simon Rippon’s “Imposing options on people in poverty: The harm of a live donor organ market”. *Journal of Medical Ethics* 40(3): 152–153. 23087186 10.1136/medethics-2012-100645

[CR8] Rippon, S. 2014. Imposing options on people in poverty: The harm of a live donor organ market. *Journal of Medical Ethics* 40(3): 145–150. 22745109 10.1136/medethics-2011-100318

[CR9] Rippon, S. 2017. Organ markets and disrespectful demands. *International Journal of Applied Philosophy* 31(2): 119–136.

[CR10] Satz, D. 2010. *Why some things should not be for sale: The moral limits of markets*. Oxford Political Philosophy. New York: Oxford University Press.

[CR11] U.S. Bureau of Labor Statistics. 2020. Census of Fatal Occupational Injuries (CFOI), Hours-based fatal injury rates by industry, occupation, and selected demographic characteristics 2020. Accessed July 29, 2024.

[CR12] Walsh, A. 2014. Commentary on Simon Rippon, “Imposing options on people in poverty: The harm of a live donor organ market”. *Journal of Medical Ethics* 40(3): 153–154. 22751956 10.1136/medethics-2012-100646

